# Longitudinal development of hand use in children with unilateral spastic cerebral palsy from 18 months to 18 years

**DOI:** 10.1111/dmcn.15370

**Published:** 2022-07-28

**Authors:** Ann‐Christin Eliasson, Linda Nordstrand, Magnus Backheden, Marie Holmefur

**Affiliations:** ^1^ Department of Women's and Children's Health, Karolinska Institutet Stockholm Sweden; ^2^ Department of Neurobiology, Care Sciences and Society, Karolinska Institutet Stockholm Sweden; ^3^ Department of Leaning, Informatics, Management and Ethics, Karolinska Institutet Stockholm Sweden; ^4^ School of Health Sciences Örebro University Örebro Sweden

## Abstract

**Aim:**

To describe the development of the use of the affected hand in bimanual tasks in children with unilateral cerebral palsy (CP) from 18 months to 18 years. Specifically, whether early development can be confirmed in a larger cohort and how development progresses during adolescence.

**Method:**

In total, 171 participants (95 males, 76 females; mean age 3 years 1 month [SD 3 years 8 months], range 18 months–16 years at inclusion) were classified in Manual Ability Classification System (MACS) levels I (*n* = 41), II (*n* = 91), and III (*n* = 39). Children were assessed repeatedly (median 7, range 2–16 times) with the Assisting Hand Assessment: in total 1197 assessments. Developmental trajectories were estimated using a nonlinear mixed effects model. To further analyse the adolescent period, a linear mixed model was applied.

**Results:**

The developmental trajectories were different between participants in MACS levels (MACS I–II, II–III) in both rate (0.019, 95% confidence interval [CI] 0.006–0.031, *p* = 0.034; 0.025, 95% CI 0.015–0.037, *p* < 0.001) and limit (19.9, 95% CI 16.6–23.3, *p* = 0.001; 7.2, 95% CI 3.3–11.2, *p* < 0.003). The individual variations were large within each level. The developmental trajectories were stable over time for all MACS levels between 7 and 18 years (*p* > 0.05).

**Interpretation:**

Children and adolescents with unilateral CP have considerable development at an early age and a stable ability to use their affected hand in bimanual activities from 7 to 18 years in all MACS levels.

## Graphical abstract

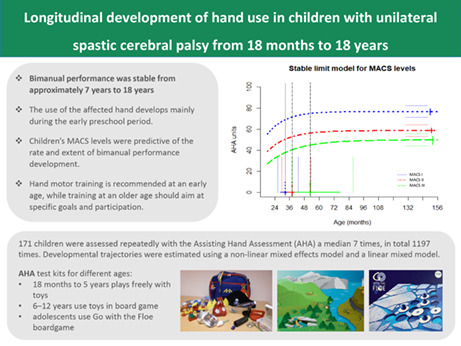

**Abbreviations**
AHA: Assisting Hand Assessment
MACS: Manual Ability Classification System

What this paper adds

- The use of the affected hand develops mainly during the early preschool period.
- Bimanual performance was stable from approximately 7 years and during adolescence.
- Children's Manual Ability Classification System (MACS) levels were predictive of the rate and extent of bimanual performance development.
- Children in MACS level III reached their stable performance at the oldest age.
- Hand motor training is recommended at early preschool period.
- The content of training for older children should aim at specific goals and participation.

Unilateral cerebral palsy (CP) is the most common type of CP, affecting approximately 40% of those with CP.^1^, ^2^, ^3^ People with unilateral CP typically have difficulties performing activities that require the use of both hands, which affects their everyday life. Bimanual performance is strongly and positively associated with independence in self‐care.^4^, ^5^ To predict children's future ability to use the affected hand in bimanual activities and to organize interventions for these children throughout childhood, more must be learned about long‐term development.

Previous studies have described the longitudinal development of hand function in children with unilateral CP in the age range from 18 months to 13 years.^6^, ^7^, ^8^ When bimanual performance measured using the Assisting Hand Assessment (AHA) was investigated, substantial development was seen in the preschool period. The higher functioning children (those classified in Manual Ability Classification System [MACS) level I) achieved the highest AHA score compared with those in other MACS levels and reached 90% of their limit (age‐90) at an earlier age (33–36 months) than children in MACS level II (38–48 months). Children in MACS level III had the lowest ability but continued to develop over the longest age period (up to 53–92 months of age). Thereafter, the ability became stable until 13 years of age for children in all three MACS levels.^6^, ^7^, ^8^ Developmental trajectories have also been studied in other areas of function, such as gross motor function,^9^ self‐care, and mobility.^10^, ^11^ In these areas, the children also demonstrate substantial development at an early age. However, the trajectory profiles vary depending on the type of ability, thereby highlighting the need for specific investigation into each type of ability.

There have been discussions about potentially reduced function at an older age. Gross motor function has been found to decrease during adolescence for those classified in Gross Motor Function Classification System (GMFCS) levels III to V.^9^ Regarding hand function, one study from Belgium that followed children over a 5‐year period (mean age at inclusion 9 years 11 months) demonstrated decreased AHA scores over time.^12^ However, no decreasing trend was observed in children up to 13 years of age in Norwegian and Swedish longitudinal studies.^7^, ^8^ Additionally, no decrease in AHA scores was found in smaller studies of adolescents and young adults with unilateral CP. In a 6‐year follow‐up after intensive training (age at inclusion 8–17 years), there were no significant changes in the AHA score, either directly after training or 6 years later. At the 10‐year follow‐up after hand surgery, the AHA score was not significantly different from that before surgery (mean age at inclusion 11 years 4 months), although an improvement was seen 6 months after surgery.^13^ Thus, the existing research has not provided conclusive evidence about the developmental trajectories in adolescents.

The aim of this study was to describe the development of the use of the affected hand in bimanual tasks in children with unilateral CP from 18 months to 18 years of age by repeated measures of the AHA and, more specifically, whether early development can be confirmed in a larger cohort and how development progresses during adolescence.

## METHOD

### Design

The study had an explorative prospective longitudinal design.

### Participants and recruitment

A convenience sample of 171 participants with spastic unilateral CP was included. The inclusion criteria were a diagnosis of spastic unilateral CP, age 18 months to 16 years at inclusion, and willingness to participate in at least two data collection points over a minimum of 12 months.

Participants (*n* = 96) were recruited from a previous longitudinal study;^7^ of these, 36 were also included in the first longitudinal study of this cohort.^6^ For the present study, another 75 participants were added to the cohort, who were recruited by their occupational therapists at local habilitation centres or children's hospitals in Stockholm County. Their demographic data are described in Table 1.

**TABLE 1:** Demographic data of the participants (*n* = 171)

Characteristic	
Number of AHA assessments for the 171 participants	1197
Mean gestational age (SD, range), weeks	36.4 (5.26, 23–43)
Born at term, *n* (%)	89 (52)
Born < 37 weeks, *n* (%)	43 (36)
Missing, *n* (%)	39 (22)
Sex, *n* (%)	
Female	76 (56)
Male	95 (44)
Affected hand, *n* (%)	
Right	93 (54)
Left	78 (46)
Mean (SD) age at recruitment, years:months	3:1 (3:8)
Median age (range) at recruitment, years	2 (1–16)
Median age (range) at last assessment, years	11 (2–19)
Median time (range) within project, years	8 (1–17)
Median number (range) of assessments	7 (2–16)
Assessment in different ages, *n* (%)	
18 months to 2 years	107 (9)
3 years	351 (29)
4 years	115 (10)
5–6 years	171 (14)
7–8 years	129 (11)
9–10 years	102 (9)
11–12 years	83 (7)
13–14 years	55 (5)
15–16 years	49 (4)
17–18 years^a^	35 (3)
Manual Ability Classification System level,^b^ *n* (%)	
I	41 (24)
II	91 (53)
III	38 (22)
Missing	1 (0.5)
Intensive intervention or hand surgery, *n*	
Constraint‐induced movement therapy	118
Surgery of upper limb	9
Both surgery and constraint‐induced movement therapy	8

Abbreviation: AHA, Assisting Hand Assessment.

^a^ The two assessments at age 19 years are included in the 17–18‐year age group.

^b^ Measured at final assessment.

The study was approved by the Ethical Review Board at Karolinska Hospital in Stockholm and the Stockholm Regional Ethical Review Board (Dnr: 03–151, 148–31, 278–32, 2281–32). The study was conducted in accordance with the ethical principles in the Declaration of Helsinki. Families signed an informed consent form at the time of recruitment, and oral consent was obtained from parents and children before each data collection point.

### Habilitation services in Sweden

All participants had full access to the children's habilitation services that are typically available in Sweden, including regular check‐ups and interventions during the study period. However, the amount of contact usually varies over the years; typically, it is more frequent at younger ages than at older ones. During the preschool years, the families have regular contact, from every second week to a few times each semester. At school age, children typically have much less contact, and for many adolescents, it is approximately once or twice a semester. The contact is typically focused on supporting and supervising the child, parents, and teachers. The treatment approach used is individualized and goal‐oriented, focusing on functional skills necessary in daily life. Intensive periods of training, such as constraint‐induced movement therapy, are mainly performed with infants and preschool children. Botulinum neurotoxin A is possible when found suitable.

### Instrumentation

#### AHA

The AHA is an observational assessment investigating how efficiently persons with a unilateral motor disability use their affected hand in bimanual activities.^14^ Age‐appropriate test activities were used: at ages 18 months to 5 years, the children played freely with the toys in the AHA test kit (Small‐kids AHA); at ages 6 to 12 years, they played a board game that involved the same toys (School‐Kids AHA); and adolescents used the board game activity ‘Go with the Floe’ (Ad‐AHA).^14^ Observed performance was rated from videotapes of the AHA sessions on a 4‐point rating scale on 20 items (AHA 5.0). The total raw score was transformed to AHA units with a range of 0 to 100 using Rasch analysis which included a logarithmic transformation, where a higher score indicated better performance. The AHA shows evidence of good intra‐ and interrater reliability as well as alternative form reliability between Small‐kids and School‐Kids AHA^15^ and School‐Kids AHA and Ad‐AHA respectively.^16^

#### MACS

The MACS describes how children with CP handle objects in everyday life on a five‐level scale, where level I indicates the best performance.^17^ Children with unilateral CP function in levels I to III, since extensive help that is needed for levels IV and V is not needed when one hand is well functioning. The mini‐MACS was used for children younger than 4 years at the last assessment. Interrater reliability between occupational therapists is excellent.^18^

### Data collection

The participants were assessed with the AHA approximately once a year up to about 12 years and every second year thereafter or sometimes less frequently. The variation in assessment regularity depended on the families' interest and opportunity to be involved in the data collection. The AHA sessions were conducted by occupational therapists at local habilitation centres or by the research team. The assessments were mostly rated by our research team, and all raters were certified AHA raters who were not aware of the child's previous results. Since the interrater reliability of the AHA is excellent (intraclass correlation coefficient = 0.97), different raters can be used with a small risk of rater bias.^15^ MACS/Mini‐MACS data were collected by the occupational therapist and updated at the same time as the AHA was collected, as well as information about hand surgery and intensive hand training. The classification level at the last measurement occasion was used in the analysis.

### Data analysis

Children's demographic characteristics were summarized using percentages, means and standard deviations, and ranges as appropriate. Developmental trajectories were estimated using both nonlinear and linear mixed effects models. First, to confirm previous results on early development in this larger sample, developmental trajectories were estimated using a nonlinear mixed effects model, which was also used in previous longitudinal studies of this cohort.^6^, ^7^ The children were grouped by MACS level, and separate development curves for each level were produced. A stable limit model was applied to the data, which was based on a negative exponential function.^19^ The negative exponential function assumes development where the hand function increases with a high rate at low age, which decreases with time, and levels out at a stable limit of development (AHA score = asymptote × [1 − e^−rate × age^]). The stable limit model results in two primary parameters: (1) rate, to capture increased performance with age, and (2) limit, to capture the estimated maximum (asymptote) AHA level that is reached at older ages. The children were expected to vary in their developmental rate and limit, but all were assumed to have 0 AHA units at birth. The parameters rate and limit were included as random effects in the model and were assumed to correlate. A mean limit and mean rate were estimated for participants in each MACS level, included as fixed effects in the model, and were compared, with a *p*‐value less than 0.05 considered to represent a statistically significant difference. The rate of development was transformed to age‐90, the age at which participants reached 90% of their limit.^6^ Approximate 95% confidence intervals and 50% interquartile ranges were calculated for limit, rate, and age‐90, on the basis of the assumption of a normal distribution of fixed effects parameters in the mixed model. A peak‐and‐decline model was investigated, but the data did not fit the model.^9^ The nonlinear models were fitted with the statistical software R version 4.0.3 (R Foundation for Statistical Computing, Vienna, Austria).

To further explore how development progresses in adolescent years, a linear mixed model with random intercept and random slope was used. The g‐matrix representing the correlation between the random intercept and random slope was set to an unstructured model. The age‐variable was centred on the age mean. In the fixed model intercept, centred age and trend components up to the sixth power of centred age were included, as well as the three MACS levels and interactions between MACS and the linear trend component of centred age. Trend components up to the third power of centred age were included as random effects in the model. All of the components that were of relevance or statistically significant were included in the statistical model. In the analysis, tests of differences between mean scores were done, and 95% confidence intervals were calculated. Comparisons across MACS levels at certain ages, such as 7, 12, 16, and 18 years, were performed. MACS level II was set as the reference category. To test changes between means, within MACS levels and across certain ages, the data were classified into 18 age classes, each representing the actual age in years, each with the range of one year and the actual year as the middle point. To analyse differences between all ages in relation to the reference age (7, 12, and 16 years), three different random intercept and random slope models were applied to the first, second, and third age‐group differences respectively. The necessary trend and group components were included in the fixed model, and the necessary random components were included in the random model for the three statistical analyses. Residual analysis was done to check the model assumptions related to each statistical analysis. The statistical software used for the linear model was SAS version 9.4 (SAS Institute Inc., Cary, NC, USA).

## RESULTS

The 171 participants were assessed with the AHA on 1197 occasions in the age range 18 months to 18 years, except two participants who were 19 years at their last assessment. There was a median of seven assessments per participant (range 2–16) over a mean period of 8 years (range 1–17). The participants were distributed over the MACS levels I–III (Table 1). A large proportion of the participants (*n* = 136) were included before 4 years of age. The observations were distributed over the studied age span (Table 1). Many participants (*n* = 118) had been involved in an intensive training programme, for example constraint‐induced movement therapy and/or hand surgery (Table 1).

### Early development and stability using the nonlinear mixed model

**FIGURE 1:**
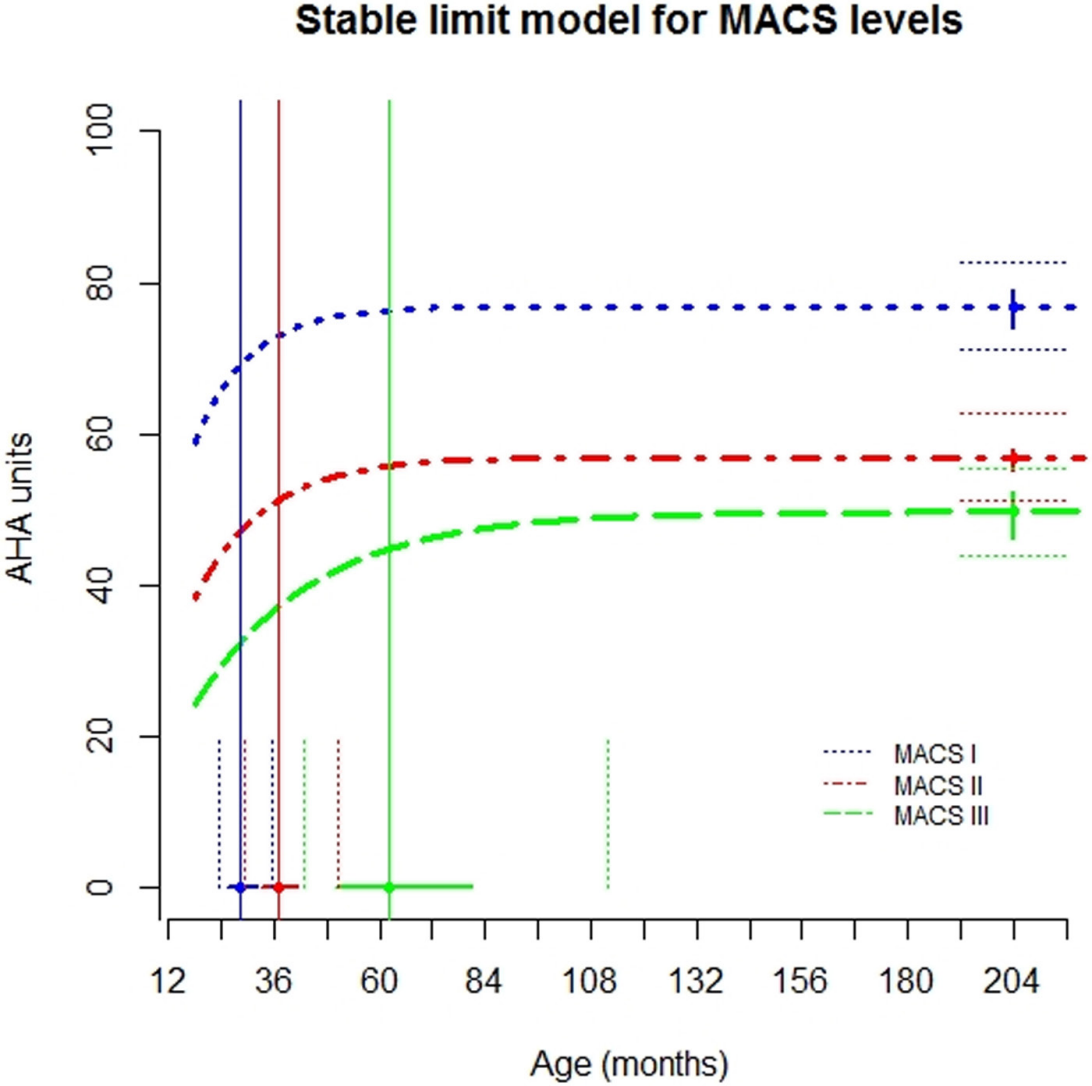
Trajectories for mean development of hand function among children from 18 months to 18 years, using the stable limit nonlinear model for Manual Ability Classification System (MACS) levels I–III, reporting 95% confidence intervals (solid lines) and 50% interquartile range (IQR) (dotted line). Blue, MACS level I; red, MACS level II; green, MACS level III. Solid vertical line: age‐90 for each MACS level. The vertical dotted lines refer to IQR for age‐90 and the horizontal dotted lines is for the limit.

The developmental trajectories for each MACS level were constructed (Figure 1 and Table 2). The stable limit model converged, demonstrating that development patterns in the cohort showed an increased development rate in younger ages. There was a significant difference in development between the three MACS level groups (Table 3). The limit for MACS level I (mean 76.9 AHA units) was significantly higher than the limit for MACS level II (mean 57.0 AHA units, *p* < 0.001), which, in turn, was significantly higher than the limit for MACS level III (mean 49.8 AHA units, *p* = 0.003). The rate of development also differed significantly between participants in MACS levels I and II (*p* = 0.034), as well as II and III (*p* < 0.001) (Tables 2 and 3). Participants in MACS level I reached their age‐90 at an average age of 28 months compared with 37 months for children in level II and 62 months for children in level III. Thus, the rate of development was faster for MACS levels I and II than for level III (Table 2).

**TABLE 2:** Parameter estimates, confidence interval (CI), and interquartile range (IQR) for the stable limit model for development of hand function measured with the Assisting Hand Assessment (AHA) for the different Manual Ability Classification System (MACS) levels

		Stable limit model							
		Limit (AHA units)			Rate		Age‐90 (months)		
MACS level^a^	*n*	Parameter estimate	95% CI	50% IQR	Parameter estimate	95% CI	Parameter estimate	95% CI	50% IQR
I	41	76.9	74.1–79.7	71.1–82.6	0.081	0.071–0.091	28	25–33	24–36
II	91	57.0	55.1–58.9	51.3–62.7	0.062	0.055–0.069	37	33–42	29–50
III	38	49.8	46.3–53.2	44.0–55.5	0.037	0.028–0.046	62	50–81	43–112

^a^ Missing data *n* = 1.

**TABLE 3:** Comparison between limits and rates for Manual Ability Classification System (MACS) levels I–III

Comparison	Difference between groups	SEM	95% CI	*p*
Limit (AHA units)				
MACS levels I–II	19.9	1.71	16.6–23.3	<0.001
MACS levels II–III	7.2	2.01	3.3–11.2	0.003
Rate				
MACS levels I–II	0.019	0.006	0.006–0.031	0.034
MACS levels II–III	0.025	0.006	0.015–0.037	<0.001

Abbreviations: AHA, Assisting Hand Assessment; CI, confidence interval; SEM, standard error of measurements.

### Development patterns during the adolescent period using the linear mixed model

**FIGURE 2:**
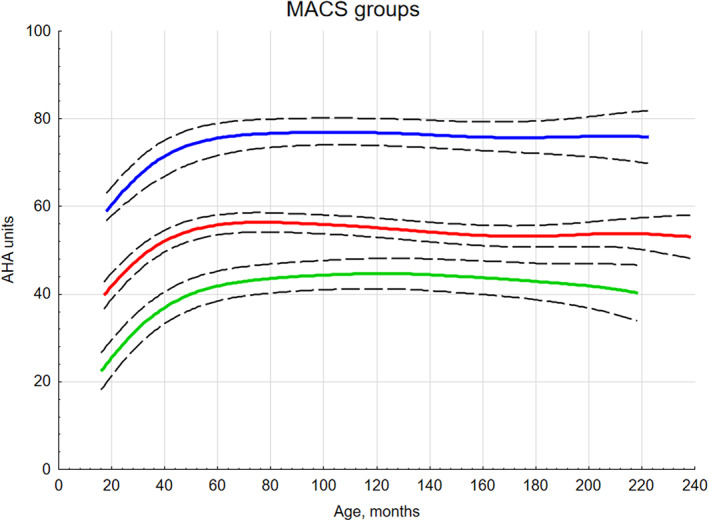
Trajectories for mean development of hand function among children from 18 month to 18 years, using the linear model for Manual Ability Classification System (MACS) levels I–III. Predicted line: blue, MACS level I; red, MACS level II; green, MACS level III reporting 95% confidence interval (dotted lines).

To further investigate whether development was stable during the period between 7 and 18 years, as suggested in the stable limit model above, the linear mixed model was applied to the whole data set. Figure 2 shows minor variations in AHA scores within MACS levels once children reached approximately 7 years of age. As with the nonlinear model, a difference in performance was seen between MACS levels I and II and II and III (*p* < 0.001 for both comparisons). When investigating the estimated mean AHA units for the four ages (7, 12, 16, 18 years), there were no major changes between the ages in AHA units for the different MACS levels (Table 4).

**TABLE 4:** Mean parameter estimates and 95% confidence intervals (CI) at different ages for the linear mixed model of the development of hand function measured with the Assisting Hand Assessment (AHA)

Age group	MACS level I		MACS level II		MACS level III	
	Mean AHA units	95% CI	Mean AHA units	95% CI	Mean AHA units	95% CI
7 years	76.6	73.4–79.9	56.2	54.0–58.4	43.4	40.1–46.7
12 years	75.9	72.6–79.2	55.5	53.2–57.8	42.7	39.3–46.1
16 years	75.1	71.6–78.7	54.7	52.0–57.3	41.9	38.3–45.5
18 years	74.9	70.8–78.9	54.4	51.1–57.7	41.6	37.5–45.7

Abbreviation: MACS, Manual Ability Classification System.

**FIGURE 3:**
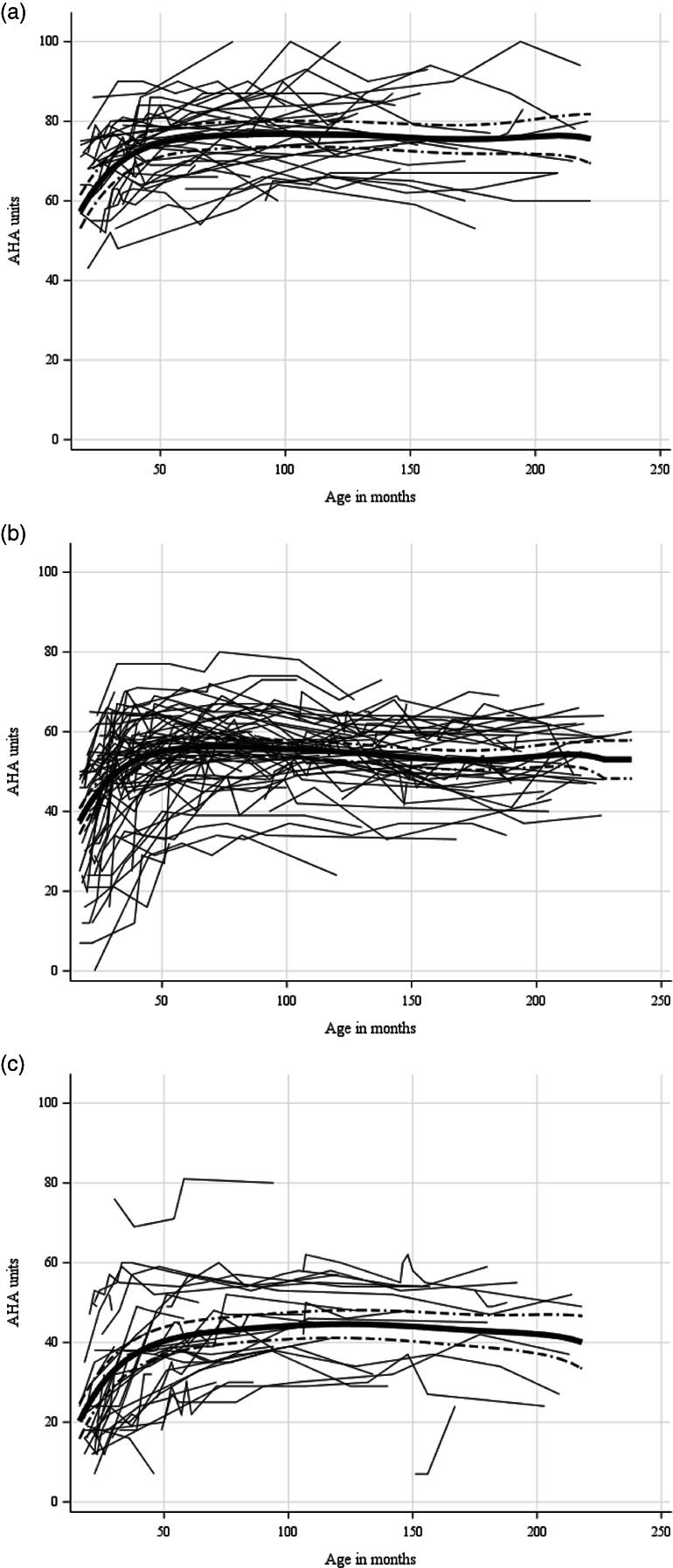
Individual developmental trajectories for children in each Manual Ability Classification System level: (a) level I; (b) level II, (c) level III. The bold solid line represents mean development calculated with the linear model; dotted lines represent the 95% confidence interval. Abbreviation: AHA, Assisting Hand Assessment.

For the sample as a whole, a slight, non‐significant decrease in bimanual performance was found between 7 and 12 years of age (−1.5 AHA units, *p* = 0.084), which continued to decrease to −2.0 units (*p* = 0.180) at 16 years of age and returned to −1.0 units at 18 years of age (*p* = 0.654) (Table 5). Mean developmental trajectories for each MACS level and individual trajectories for each child are found in Figures 2 and 3 respectively. Although the performance was stable over ages, large individual variation was found in each MACS level.

**TABLE 5:** Comparison between estimates of means and 95% confidence intervals (CI) at different ages and Manual Ability Classification System levels I–III

Comparison	Difference in AHA units between groups	95% CI		*p*
		Lower	Upper	
7–12 years	−1.5	−3.1	0.2	0.084
7–16 years	−2.0	−5.1	1.0	0.180
7–18 years	−1.0	−5.8	3.8	0.654
12–16 years	−0.9	−3.2	1.3	0.402
12–18 years	0.0	−3.5	3.5	0.997

Abbreviation: AHA, Assisting Hand Assessment.

## DISCUSSION

The development patterns found in the present study confirm the findings from the previous study,^7^ now in a considerably larger sample. The children with unilateral CP increased their ability to use their affected hand in bimanual activities during the preschool period to a significantly different extent between MACS levels. The current study also shows that bimanual performance is stable from approximately 7 to 18 years of age for children/adolescents in all MACS levels.

Although the general patterns were the same as in the previous study, minor differences were detected, which were that children classified in MACS level I reached age‐90 on average 5 months earlier, children functioning in MACS level II had negligible difference (2 AHA units lower stable limit and 1 month's longer age‐90), and children in MACS level III had a 9‐month longer period of development in the current study. The results are in agreement with the Norwegian study on a younger cohort (mean age at last assessment 6 years).^8^ They showed similar characteristics of the trajectories for participants in all MACS levels while the overall level of functioning was somewhat higher. The largest difference was found for children functioning in MACS level II, where the Norwegian children scored 11 AHA units higher and age‐90 occurred 30 months later than the current study. The differences might depend on different recruitment procedures. The Norwegian study was population‐based, although the drop‐out rate was high: only 25% of the children with unilateral CP had repeated AHA measures and thereby participated.^8^ The current study was hospital‐based and typically included children with more severe impairments. Both studies had fewer children in MACS level I than previous reports in a population‐based Swedish study.^20^ One could speculate that the stable limit should probably be somewhat higher for MACS level I since the children with mild impairments are probably missing owing to minimal contact with the rehabilitation services.

To further investigate the stability of the developmental progress during adolescence, a second statistical model was used. In the linear mixed model, the shape of the trajectories was based on data and not on an assumed development. Despite minor fluctuations, this model also supports a stable performance in all MACS levels throughout adolescence from approximately age 7 years. The stability of performance over ages found in Scandinavian studies differs from a Belgian study where AHA scores decreased over a 5‐year period; on the other hand, the unimanual capacity and perceived ability increased in the same study, making it difficult to interpret the result.^12^ In addition to different recruitment procedures, personal factors and previous treatment strategies probably influence the future developmental trajectory of hand use, and together with different geographical locations and social contexts, generalizability becomes unreliable.

For a greater understanding of the children's and adolescents' life situation, longitudinal development needs to be described from different perspectives of functioning. Although self‐care was strongly and positively associated with bimanual performance (AHA) when self‐care was investigated by the Paediatric Evaluation of Disability Inventory‐Computer Adaptive Test,^5^ there were important differences. Children functioning in MACS level I developed their bimanual performance (AHA) during the shortest period compared with those in MACS level III, while self‐care developed in an opposite pattern where children in MACS level III had the shortest period for development of self‐care.^4^, ^11^ There were also differences in development between manual ability and gross motor function.^17^ Children functioning in GMFCS levels I and II need a longer time to achieve their maximum development than those in GMFCS levels IV and V, namely opposite to the development of bimanual performance (AHA). In summary, the developmental trajectories are different for the different types of ability and for children classified in different functional levels.

The recruitment procedure was a limitation in this study, in which age distribution, severity level, and environment were crucial points for external validity and generalizability. In this convenience sample, there were more children in the younger age groups and fewer in the older ones, meaning the estimates were less reliable in the older age groups. As in previous studies, there was considerable variation in the AHA scores within each MACS level. Extreme AHA scores might be related to the complexity of the diagnosis and occurrence of associated impairments affecting daily life and thereby the choice of classification level.

There was also a large variation in the number of assessments per child, ranging from 2 to 16. For longitudinal analyses, more repeated assessments are an advantage. Three assessments are commonly recommended, which was fulfilled for most of the children in this study; only 12 children had two assessments. When Klevberg et al. compared two and three repeated assessments, it had no effect on the model;^8^ this is why the 12 children were kept in the model.

Over the years, some children were included in specific interventions, such as constraint‐induced movement therapy, hand surgery, and botulinum neurotoxin A, in addition to ordinary treatment. This was noted (Table 1) but not included in the statistical models. The long‐term effect of the specific interventions is mainly unknown.^21^ Although it is difficult to separate the effect of training from other factors in life, we believe that intervention influences development. This can be seen when compared with children living in Uganda with no access to treatment, whose developmental trajectories according to the Paediatric Evaluation of Disability Inventory showed a decline and were clearly different from children in the Western world.^22^

## CONCLUSION

Previous knowledge that the AHA score at 18 months together with the MACS levels is predictive of future development was confirmed in this larger study. Children classified as having higher ability (MACS level I) had both a higher rate and limit of development and a shorter period of development than those having a lower ability (MACS level II). Children functioning in MACS level III had the lowest limit, and development occurred during the longest time. The stable performance lasted throughout adolescence for participants in all MACS levels from approximately 7 years.

This demonstrates that, if the children have learned to use both hands in a meaningful way, they continue to use them when growing up. On an individual level, large variation in development is seen; therefore regular follow‐ups for children in all MACS levels in the clinic are important. Furthermore, the stabilizing of trajectories gives an important opportunity to shift the focus from capacity‐related intervention to goal‐directed training and participation interventions.^23^, ^24^, ^25^ There is positive evidence that children at any age and functional level can learn new skills.^26^ This perspective is also nicely described by Lidman et al. in a qualitative study where adolescents reported that they learn skills when it fits into their life situation and that it is a conscious choice whether to use one or both hands.^27^

## Data Availability

Author elects to not share data.
